# Initial Single-Center Experience with Robotic Roux-en-Y Gastric Bypass: A Retrospective Case Series

**DOI:** 10.3390/jcm14092967

**Published:** 2025-04-25

**Authors:** Antonio Vitiello, Antonio Braun, Libero Giambavicchio, Arianna Corvasce, Giovanna Berardi, Vincenzo Pilone

**Affiliations:** 1 Advanced Biomedical Sciences Department, Naples “Federico II” University, AOU “Federico II”–Via S. Pansini 5, 80131 Napoli, Italy; 2 San Pier Damiano Hospital–Faenza, Emilia-Romagna–GVM, Via Portisano, 1, 48018 Faenza, Italy; 3 Public Health Department, Naples “Federico II” University, AOU “Federico II”–Via S. Pansini 5, 80131 Napoli, Italy

**Keywords:** robotic bariatric surgery, RYGB, Hugo robotic-assisted surgery system

## Abstract

**Objective:** To evaluate the outcomes of a preliminary single-center experience with Robotic Roux-en-Y Gastric Bypass (R-RYGB) using the Hugo™ Robotic-assisted Surgery system, focusing on operative time, perioperative complications, and length of hospital stay. **Methods:** A retrospective review identified 19 consecutive patients who underwent R-RYGB with the Hugo™ system between January 2023 and January 2024. The baseline data collected were sex, age, and BMI. Key outcomes measured were operative time, docking time, length of hospital stay, conversions to open or laparoscopic surgery, reinterventions, readmissions, and intraoperative and early (<30 days) postoperative complications (bleeding, leak, and stenosis). **Results:** Nineteen patients underwent R-RYGB using the Hugo™ system. The mean age was 43.5 ± 10 years, and the mean BMI was 39.4 ± 2.9 kg/m^2^. Among these patients, 11 were female (57.9%); 3 had diabetes mellitus (15.8%), 3 had obstructive sleep apnea syndrome (15.8%), and 7 had hypercholesterolemia (36.8%). The mean operative time was 177.8 ± 34.7 min, with a mean docking time of 12.5 ± 4.6 min. The mean length of hospital stay was 3.1 ± 0.2 days. There were no intraoperative complications, conversions, reinterventions, readmissions, or 30-day postoperative complications. All procedures were completed uneventfully. **Conclusions:** Our preliminary experience suggests that R-RYGB using the Hugo™ Robotic-assisted Surgery system is safe and feasible, with acceptable operative and docking times and no perioperative complications.

## 1. Introduction

The obesity pandemic continues to spread worldwide, and metabolic and bariatric surgery (MBS) is the most effective treatment for severe obesity and associated medical problems. Laparoscopic Roux-en-Y Gastric Bypass (RYGB) is one of the most performed metabolic procedures worldwide, representing a significant portion of bariatric interventions in many countries [1]. However, bariatric surgeons have traditionally relied heavily on laparoscopic techniques due to their established feasibility and outcomes. According to the latest IFSO Report, 23% of all primary metabolic interventions in the U.S. were performed robotically. In contrast, the adoption rate of robotic surgery remains remarkably low in many European countries, such as Italy (0.6%), France (0%), and Sweden (0%) [2].

However, in Italy, the utilization of robotic bariatric surgery is continuously rising [3]. This increase is also driven by the introduction of new robotic platforms into the market. As a result, there is a significant potential to improve the effectiveness and outcomes of robotic bariatric procedures. The adoption of these cutting-edge technologies is expected to further advance the field of bariatric surgery in Italy, leading to better patient care and fostering innovation within surgical practices.

The aim of this study was to retrospectively assess the outcomes of a single-center preliminary experience with Robotic Roux-en-Y Gastric Bypass (R-RYGB) using a Hugo Robotic-assisted Surgery system with particular attention to operative time, perioperative complication rates, and length of hospital stay.

## 2. Materials and Methods

A retrospective review was performed using a prospectively maintained database at a high-volume bariatric center to identify all consecutive patients who underwent Robotic Roux-en-Y Gastric Bypass (R-RYGB) between January 2023 and January 2024. Indications for metabolic bariatric surgery (MBS) included patients aged 18 to 60 years with a body mass index (BMI) >35 kg/m^2^ or a BMI >30 kg/m^2^ in the presence of one or more obesity-related comorbidities [4]. Exclusion criteria comprised any previous bariatric or abdominal surgery and evidence of severe gastro-esophageal reflux or a hiatal hernia exceeding 2 cm, as determined by upper gastrointestinal endoscopy.

Baseline demographic and clinical data—including sex, age, and BMI—were extracted directly from the clinical records. In addition, intraoperative and postoperative data were systematically recorded. Key variables comprised operative time, length of hospital stay, and any instance of conversion to either open surgery or laparoscopy. The study protocol required detailed tracking of reinterventions and readmissions, as well as early (<30 days) postoperative complications such as bleeding, anastomotic leak, and stenosis. All data were meticulously entered into a Microsoft Excel 365™ datasheet, with routine verification protocols implemented to ensure accuracy and consistency.

### 2.1. Robotic Roux-en-Y Gastric Bypass (R-RYGB) Surgical Technique Using Hugo™

**Figure 1:**
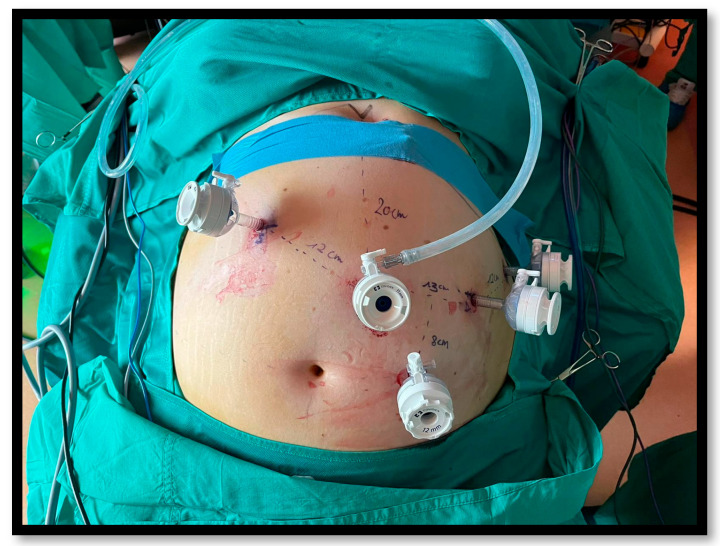
Trocars position.

Under general anesthesia, patients are placed in the supine position with legs split (“French” position), ensuring padding of pressure points and securing the body with a gel pad and straps to prevent shifting in the reverse Trendelenburg position. Trocar placement begins with a 12 mm camera port in a supraumbilical position, slightly left of the midline, approximately 15–20 cm below the xiphoid process. After establishing pneumoperitoneum, three 8 mm robotic trocars are inserted: one on each flank and one in the left subcostal space. An additional 12 mm accessory trocar for the assistant is placed inferiorly, between the camera trocar and the surgeon’s right-hand trocar (Figure 1).

The Hugo™ system (Medtronic, Minneapolis, MN, USA), consisting of four independent arm carts, is docked with configurations tailored to the patient’s body type. The robotic arms are equipped with a bipolar fenestrated grasper for the left hand, monopolar curved scissors for the right (switched to a needle driver during anastomosis), and a secure Cadiere forceps or double fenestrated grasper for the fourth trocar.

The procedure starts with the creation of the gastric pouch by entering the lesser sac along the lesser curvature about 6 cm from the gastroesophageal junction. The laparoscopic linear stapler (Signia™-Medtronic, Minneapolis, MN, USA) is introduced through the accessory trocar, creating the pouch using a 38 F orogastric bougie for calibration.

A small bowel loop, approximately 75 cm distal, is brought up in an antecolic fashion for the first anastomosis, which is a robotic, hand-sewn, end-to-side gastrojejunal (GJ) anastomosis performed with two layers of running V-Loc™ (Medtronic, Minneapolis, MN, USA) sutures. For the second anastomosis, another bowel loop, 75–100 cm distal from the GJ anastomosis, is anastomosed side-to-side with the afferent limb using a single bronze cartridge. This double-loop technique ensures effective rerouting of the gastrointestinal tract.

The insertion holes are closed with running absorbable sutures (V-Loc™). The integrity of the anastomoses is verified using a blue methylene and pneumatic test. The final step involves creating the Roux-en-Y by dividing the jejunum between the anastomoses with a linear stapler (single bronze cartridge). A drain is placed posteriorly to the GJ anastomosis.

### 2.2. Postoperative Protocol

A standardized postoperative protocol, tailored specifically for bariatric patients, was implemented. Patients were kept nil per os (NPO) until the second postoperative day. Routine complete blood examinations and blood counts were conducted on the first postoperative day for all patients. Discharge criteria included the absence of clinical complications or postoperative biochemical and imaging abnormalities, successful tolerance of oral alimentation, independence in activities of daily living, and patient acceptance of discharge.

## 3. Results

Our search found a cohort of 19 patients who underwent R-RYGB with Hugo with a mean age of 43.5 ± 10 years and a mean body mass index (BMI) of 39.4 ± 2.9 kg/m^2^. Among these patients, 11 were female (57.9%), 3 had diabetes mellitus (15.8%), 3 were diagnosed with obstructive sleep apnea syndrome (OSAS; 15.8%), and 7 presented with hypercholesterolemia (36.8%). The surgical procedures were performed with a mean operative time of 177.8 ± 34.7 min (docking time 12.5 ± 4.6 min). Postoperatively, patients experienced a mean hospital length of stay of 3.1 ± 0.2 days. There were no 30-day postoperative complications, and all cases were completed uneventfully.

## 4. Discussion

Despite the gradual global expansion of robotic surgery, significant doubts remain regarding its application in metabolic procedures. The main objections to the robotic approach include longer operative times, higher costs, and the lack of demonstrable benefits for patient recovery when compared to traditional laparoscopy.

While the evidence is still conflicting regarding the Robotic Sleeve Gastrectomy [5,6], robust data support the robotic approach for the RYGB.

Even if robotic-assisted bariatric surgery promises mechanical advantages, its outcomes have not consistently proven superior to those of laparoscopic approaches, particularly given its higher cost and potential benefits in more complex or revisional cases. This systematic review and meta-analysis aimed to compare perioperative outcomes between Robotic and Laparoscopic Roux-en-Y Gastric Bypass procedures in patients with obesity. Utilizing PRISMA guidelines, a comprehensive literature search was conducted across Embase, Medline, PubMed, Cochrane Library, and Google Scholar, ultimately including 28 studies encompassing a total of 82,155 patients—9051 undergoing robotic bypass surgery (RBS) and 73,104 undergoing laparoscopic bypass surgery (LBS). The analysis revealed that while most endpoints (complication rate, anastomotic leak, anastomotic stricture, surgical site infections, hospital readmission, length of stay, operative time, conversion rate, and mortality) were comparable between RBS and LBS, the robotic approach demonstrated a significantly higher 30-day reoperation rate (4.4% vs. 3.4%; odds ratio 1.31 [95% CI, 1.04–1.66]; *p* = 0.027; I^2^ = 43.5%). Overall, the review concluded that there is no significant difference in key outcomes between the two surgical modalities, with the robotic platform only showing a marginally increased reoperation rate [7]. 

In 2015, Economopoulos et al. [7] performed a systematic review and meta-analysis comparing Robotic-assisted versus Laparoscopic Roux-en-Y Gastric Bypass (RYGB). Analyzing 14 comparative and 11 non-comparative studies encompassing 5145 patients, they found that Robotic-assisted RYGB had comparable clinical outcomes to Laparoscopic RYGB. Specifically, the robotic-assisted approach was associated with significantly fewer anastomotic strictures, fewer reoperations, and a decreased length of hospital stay.

In 2019, Sebastian et al. [8] conducted a propensity-score-matched analysis comparing Robotic-assisted versus Laparoscopic Roux-en-Y Gastric Bypass (RYGB) using the MBSAQIP database. The study found that Robotic RYGB had significantly longer operative times (158.29 ± 65 vs. 120.17 ± 56 min; *p* < 0.001) but showed fewer requirements for blood transfusions (0.64% vs. 1.16%; *p* = 0.004) compared to Laparoscopic RYGB. When including operative time and conversion rates in the analysis, the robotic approach demonstrated a shorter length of hospital stay (2.12 ± 1.9 vs. 2.30 ± 3.1 days; *p* < 0.001) and reduced rates of anastomotic leaks, renal complications, and venous thromboembolism.

Similarly, El Chaar et al. [9] investigated outcomes of Robotic-assisted Roux-en-Y Gastric Bypass (RA-RYGB) versus Laparoscopic RYGB (L-RYGB) using the 2020 MBSAQIP database, analyzing 168,568 patients. Through propensity score matching and inverse probability treatment weighting, they found that for RYGB procedures, the incidence of transfusions was significantly lower in the RA-RYGB group compared to the L-RYGB group. There were no significant differences in the rates of Serious Event Occurrences (SEOs) or 30-day postoperative interventions between the two groups. However, operative times were significantly longer for the robotic-assisted approach.

Using MBSAQIP data from 2015 to 2021, 286,531 RYGB cases (87% LGB, 13% RGB) yielded 25,594 matched pairs comparing LGB and RGB. Mortality was low (0.1%) and similar, while RGB had lower SSI (0.9% vs. 1.3%, *p* < 0.001) and lower bleeding (0.3% vs. 0.4%, *p* = 0.04). However, RGB showed higher readmission (5.8% vs. 4.9%, *p* < 0.001), reoperation (2.2% vs. 1.85%, *p* = 0.005), and morbidity (7.6% vs. 6.8%, *p* < 0.001). Operative length was longer for RGB (*p* < 0.001); in the early cohort (2015–2018), RGB also had longer operative duration and LOS (*p* < 0.001). In the later cohort (2019–2021), SSI (*p* = 0.006) and bleeding (*p* = 0.046) remained lower with RGB, morbidity was higher (*p* = 0.005), and LOS was comparable [10].

A retrospective study evaluated the efficiency and cost-effectiveness of robotic versus laparoscopic stapling in Robotic-assisted Roux-en-Y Gastric Bypass (RYGB) procedures by analyzing 105 patients treated between May 2022 and November 2023, where 50 patients (47.6%) underwent the robotic approach and 55 (52.4%) underwent the laparoscopic technique, with approximately 40% of these cases including hiatal hernia repair. All procedures utilized a six-port approach, and the gastric pouch and anastomoses were created using either robotic SureForm™ (Intuitive, Sunnyvale, CA, USA) staplers or laparoscopic Signia™ systems, with hiatal hernia repairs conducted as indicated. Notably, the use of laparoscopic staplers significantly improved procedural efficiency, reducing operating room time from 124.24 ± 26.98 to 106.62 ± 28.97 min (*p* = 0.0017), console time from 93.98 ± 24.16 to 77.75 ± 26.52 min (*p* = 0.0015), active time from 83.68 ± 21.90 to 69.80 ± 23.24 min (*p* = 0.0022), and waiting time from 10.30 ± 4.90 to 7.95 ± 6.23 min (*p* = 0.0348). Moreover, laparoscopic staplers required fewer instrument exchanges (median 7 [IQR 5–8] versus 18 [IQR 17–21], *p* < 0.0001) and fewer reloads (median 6 versus 7, *p* = 0.0002), which translated into significantly lower total stapling costs of USD 1477 ± 89 compared to USD 2175 ± 148 (*p* < 0.0001) [11].

Most of the data on robotic bariatric procedures originate from studies employing the Intuitive DaVinci system, which has been widely acknowledged as an exceptionally effective and reliable instrument for complex surgical interventions. This system’s advanced technology—ensuring enhanced precision, flexibility, and consistent outcomes—has established it as the gold standard in the field of robotic surgery. However, several authors have raised concerns regarding the potential for bias in the published literature, noting that open payments from the corporation might have influenced study results and interpretations. Parker et al. [12] examined if the robotic bariatric surgery literature is driven by robust data or by financial incentives to surgeons. They reviewed studies focusing on operative time, complication rates, and technical advantages, alongside public physician payment records. Their analysis revealed that studies by surgeons with substantial industry payments consistently reported superior outcomes. Despite increasing clinical data supporting robotic surgery, the findings suggest potential bias from financial conflicts of interest. The study calls for enhanced financial transparency and independent, rigorously designed research to evaluate robotic surgery’s true efficacy and safety.

To date, few studies have been published regarding Robotic Roux-en-Y Gastric Bypass (R-RYGB) using the Hugo Robotic-assisted Surgery system, which provides the reliability of robotic surgery combined with the safety of laparoscopic staplers.

In 2023, Raffaelli et al. [13] evaluated the feasibility of performing Roux-en-Y Gastric Bypass (RYGB) using the novel Hugo™ Robotic-assisted Surgery (RAS) system. In early 2023, four consecutive patients (two females, two males) with a median BMI of 40 kg/m^2^ (range: 36–46) underwent minimally invasive Robotic-assisted RYGB without exclusion criteria. The median docking time was 8 min (range: 7–8.5 min), and the median console time was 127.5 min (range: 95–150 min). All procedures were completed without intraoperative complications, conversions to laparoscopy or open surgery, or the need for additional ports. No early postoperative complications were observed, indicating that RYGB using the Hugo™ RAS system is feasible and safe based on this initial experience.

Another study [14] assessed the inaugural clinical application of the Hugo™ RAS robotic system for Roux-en-Y Gastric Bypass in 15 patients and demonstrated that the platform is both feasible and safe, with no significant intraoperative or postoperative complications. A notable short learning curve was observed as operative times steadily improved across cases, and the outcomes achieved were consistent with globally accepted standards for robotic surgery. The research further suggested that experienced surgeons can efficiently transfer their skills from other robotic platforms. While these initial findings are promising, the authors underscored the necessity for larger-scale studies to validate and extend these conclusions.

The same authors [15] also performed a retrospective study comparing the Hugo™ Robotic-assisted Surgery system (Hugo™-RAS) and the DaVinci^®^ Surgical System (DaVinci^®^-SS) in Robotic Roux-en-Y Gastric Bypass (RYGB) procedures. Reviewing bariatric surgeries from January 2013 to December 2023, they identified 135 patients (90 DaVinci^®^-SS and 45 Hugo™-RAS). After propensity score matching, each group comprised 45 patients matched on age, gender, BMI, comorbidities, and past abdominal operations. The study found no significant differences between the two platforms in early postoperative complications (*p* = 1), mean operative times (docking time *p* = 0.176, console time *p* = 0.678, total operative time *p* = 0.229), or hospital stay duration (*p* = 0.052). The results indicate that both Hugo™-RAS and DaVinci^®^-SS offer comparable safety profiles and effectiveness for Robotic RYGB. While DaVinci^®^-SS is more widely adopted, this study highlights the potential of Hugo™-RAS as an effective solution in robotic bariatric surgery.

The cumulative evidence from previous studies is consistent with our findings, demonstrating that Robotic Roux-en-Y Gastric Bypass (R-RYGB) is a feasible and safe procedure with acceptable docking times. While our results indicate that R-RYGB can be performed effectively, we observed that operative times may be longer during the learning curve compared to laparoscopic approaches. This suggests that although the robotic approach maintains patient safety and does not significantly prolong surgery in the long term, the initial operative time can be a consideration during the early adoption phase.

Moreover, the successful completion of all cases without intraoperative complications or conversions to laparoscopic or open surgery underscores the reliability of the robotic platform in bariatric procedures. The absence of early postoperative complications further validates the safety profile of R-RYGB. These findings suggest that the integration of robotic systems into bariatric surgery enhances surgical precision and may offer improved outcomes for patients with obesity.

Our study adds to the growing body of evidence supporting the use of robotic assistance in complex bariatric surgeries. The demonstrated feasibility and safety, along with acceptable docking times, highlight the potential benefits of adopting Robotic Roux-en-Y Gastric Bypass as a standard practice in bariatric surgical centers, which could be extended also to all upper GI surgery [16]. While the operative time may initially be longer during the learning curve, this challenge appears to be manageable and outweighed by the advantages offered by the robotic platform. This advancement aligns with global trends and may contribute to improved patient care and surgical innovation in the field of metabolic surgery.

## 5. Conclusions

In conclusion, our initial experience indicates that the Hugo™ Robotic-assisted Surgery system is a promising platform for performing complex minimally invasive procedures such as Roux-en-Y Gastric Bypass (RYGB). We demonstrated its safety and feasibility, highlighting advantages like enhanced precision and dexterity in anastomosis construction. The main limitation observed was the increased operative time, which is probably also associated with higher costs.

Our experience with Robotic Roux-en-Y Gastric Bypass (R-RYGB) demonstrates several significant advantages of the robotic platform. The system’s articulated instrument tips and high-definition three-dimensional imaging facilitate precise dissection and enable meticulous execution of the procedure. Enhanced visualization improves the surgeon’s ability to navigate complex anatomical structures, thereby contributing to greater surgical accuracy.

Improved ergonomics constitute another key benefit. The ergonomic design of the robotic console minimizes surgeon fatigue, a critical factor when multiple procedures are performed within a single operative day. This ergonomic efficiency not only enhances performance but also contributes to sustained surgical precision over prolonged periods.

Furthermore, the robotic approach effectively addresses the technical challenges associated with critical operative tasks, such as suturing and the construction of two anastomoses. These maneuvers require a level of dexterity that is often compromised in traditional laparoscopic techniques. With the assistance of robotic technology, these tasks are executed with enhanced control and consistency, potentially reducing the incidence of technical complications.

Moreover, R-RYGB is frequently performed concomitantly with additional interventions, including abdominal hernia repair and cholecystectomy. In these combined procedures, the robotic platform streamlines the operative workflow and optimizes overall surgical performance.

However, larger-scale studies are necessary to validate these early impressions and draw definitive conclusions about their efficacy and impact on bariatric surgery. Further research will help determine the system’s potential to improve surgical outcomes and advance the field.

## Data Availability

Data can be obtained from the corresponding author upon request.
